# LSCD-Pose: A Feature Point Detection Model for Collaborative Perception in Airports

**DOI:** 10.3390/s25103176

**Published:** 2025-05-18

**Authors:** Ruifeng Meng, Jinlei Wang, Yuanhao Huang, Zhaofeng Xue, Yihao Hu, Biao Li

**Affiliations:** 1 School of Aviation, Inner Mongolia University of Technology, Hohhot 010021, China; 2 State Key Lab of Intelligent Transportation System, Beihang University, Beijing 100191, China; 3 School of Transportation Science and Engineering, Beihang University, Beijing 100191, China

**Keywords:** collaborative perception, YOLOv8-Pose, coordinate-conversion, LSCD-Pose, computing resources

## Abstract

Ensuring safety on busy airport aprons remains challenging, particularly in preventing aircraft wingtip collisions. In this study, first, a simplified coordinate mapping method converts pixel detections into accurate spatial coordinates, improving aircraft position and velocity estimates. Next, an innovative dynamic warning area with a classification mechanism is introduced to enable faster responses from airport staff. Finally, this study proposes LSCD-Pose, a real-time detection network enhanced by lightweight shared modules, significantly reducing model size and computational load without sacrificing accuracy. Experiments on real airport datasets representing various apron scenarios demonstrate frame rates up to 461.7 FPS and a 90.5% reduction in model size compared with the baseline. Visualizations confirm the solution’s versatility and efficiency in effectively mitigating wingtip collisions and enhancing apron safety.

## 1. Introduction

The apron is a designated area at terrestrial airports for accommodating aircraft engaged in passenger, tourist, mail, or cargo loading and unloading, as well as refueling, parking, or maintenance [1]. This environment is highly dynamic; aircraft frequently traverse the apron for takeoff and landing, while ground support equipment—such as baggage carts, fuel trucks, and catering vehicles—also operates in the same vicinity. Because aprons are often crowded and aircraft are parked in close proximity, they are susceptible to scraping incidents. According to research data [2], the global cost of unreported apron activities leading to aircraft damage, maintenance requirements, flight cancellations, or delays exceeds USD 10 billion annually. Recent analyses indicate that collisions involving ground service equipment (GSE) account for approximately one-quarter of apron damage events [3], while aircraft-to-aircraft contact (e.g., wingtip-to-wingtip collisions) accounts for roughly 10%. For example, one ramp event study showed that 26% of incidents involved aircraft striking GSE or vehicles, 25% occurred during towing or pushback operations, and 10% entailed an aircraft colliding with another aircraft. Notably, the outer wingtip region is frequently identified as a collision-prone area during apron taxiing, making wingtip strikes a recurring hazard at busy airports.

In an effort to enhance the safety of aircraft operations on the tarmac, researchers employ perception methods to detect potential conflicts between aircraft. Mainstream approaches typically fall into two categories: radar-based positioning and image-based detection. Radar technology has a long developmental history and demonstrates significant adaptability. According to research and market reports published in the past two years [4], the installation ratio of airport radar systems varies by region, airport size, and type. In general, small and medium-sized airports often fail to fully deploy radar systems due to high costs and infrastructure limitations. In addition, medium and small airports frequently find the high costs associated with radar technology prohibitive, underscoring the need for a lower-cost alternative to fulfill their ground surveillance requirements.

Video-based monitoring methods [5] can overcome these limitations and provide several advantages. Current surveillance algorithms can be broadly classified into two main categories: two-stage and one-stage approaches. Two-stage algorithms initially generate candidate regions and then apply a classifier to determine whether these regions contain target objects. Representative examples of two-stage algorithms include R-CNN [6], Fast R-CNN [7], and Faster R-CNN [8]. Although these methods offer high detection accuracy, their relatively slow computational speed does not meet the real-time requirements commonly found in airport environments.

To enhance detection efficiency [9,10], current research predominantly favors the use of single-stage algorithms, which can directly process the entire image as input and run the classifier globally in one pass. This approach enables rapid and accurate identification of target objects within a short time frame. For instance, Lyu and Zhang [11] optimized and applied an improved YOLOv5s model, successfully achieving real-time, efficient monitoring in apron environments. Moreover, addressing the issue of small target object sizes in real airport scenarios, Zhou et al. [12] introduced a small target detection model called ASSD-YOLO, effectively enhancing the detection accuracy of datasets collected in actual airport environments.

Recently, advanced collaborative perception technologies [13,14] have been introduced to further augment video-based surveillance in apron areas. By integrating multi-source sensors, real-time communication, and distributed computing resources, these methods enhance detection coverage and accuracy across complex apron scenarios, complementing existing single-stage or two-stage detection algorithms.

Nevertheless, while many studies employ object detection algorithms to identify entire aircraft for conflict detection, accurately detecting wingtips—highly susceptible to collisions—remains problematic. Because wingtips share similar coloration with other sections of the wing, they are frequently mistaken for adjacent areas during detection. Environmental factors, such as fluctuating lighting, also influence detection performance. Even under varying lighting conditions, wingtips [15] often display similar shapes and contours. However, as the aircraft taxis, changes in a camera’s angle can alter the wingtips’ apparent shape, further complicating detection. Although researchers like Shen [16] have sought to improve model robustness by increasing the number of observation targets, this approach can add parameters and computational complexity, potentially reducing frame rates and hampering real-time performance.

To address these limitations, this study seeks to resolve issues of low accuracy, inefficiency, and insufficient real-time performance in apron monitoring. The research objectives are threefold: (1) to develop a standardized dataset using a sandbox model of the airport apron for comprehensive testing under diverse operational conditions, (2) to refine the YOLOv8-Pose algorithm for enhanced efficiency and accuracy in wingtip detection during active airport operations, and (3) to design a conflict detection model based on wingtip keypoint detection. By leveraging improved detection capabilities, the proposed model aims to predict potential collision points in real time, ultimately advancing overall safety on the apron.

This paper makes the following contributions:

- We first developed a straightforward coordinate conversion method that transforms pixel coordinates into actual coordinates, enabling more precise detection of conflicts occurring on the apron.
- A new network architecture was designed by integrating StemBlock, PShuffle-Block, and DSConv. This combination significantly reduces the model’s parameter count and computational complexity while almost not affecting its accuracy.
- We proposed a novel dynamic detection head for YOLOv8-Pose, which, through the use of shared convolutions, achieves model lightweighting while enhancing the detection head’s positioning and classification capabilities.

## 2. Methodology

### 2.1. Problem Definition

During taxiing on the apron, aircraft wingtips rank among the most collision-prone areas. For research purposes, this section defines a wingtip conflict as any scenario that occurs during ground operations—such as gate parking, taxiing, or runway holding—where aircraft are parked or maneuvered too closely, thus reducing the safe clearance between wingtips or between a wingtip and other critical aircraft components (e.g., the fuselage or engines). To safeguard aircraft operations, airports establish specific clearance standards for parking and minimum distance requirements for taxiing or waiting, thereby preventing wingtip collisions.

### 2.2. Coordinate Conversion

**Figure 1:**
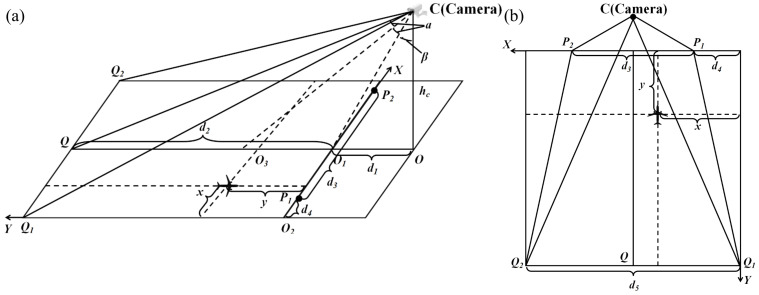
A diagram of the camera calibration approach employed in this study. (**a**) side view, (**b**) top view.

Accurate conflict detection relies on precise inter-aircraft distance calculations, which require converting pixel data into real-world 3D coordinates. This process involves camera calibration, a cornerstone of computer vision. Common calibration methods [17]—typically involving 3D objects, 2D planes, or 1D lines—often rely on planar configurations. In contrast, this study introduces a novel calibration method that bypasses conventional planar calibration, yielding high accuracy, reduced computational complexity, and simplicity. Figure 1 illustrates this innovative calibration approach.

The parameter measurements include the length of SO as $h_{c}$, OF as $d_{1}$, $O_{1}Q$ as $d_{2}$, $P_{1}P_{2}$ as $d_{3}$, and $Q_{1}Q_{2}$ as $d_{4}$. The pixel coordinates of the detection target, obtained using keypoint detection algorithms, are $\left(x_{a},y_{a}\right)$. The image has 1080 horizontal pixels and 960 vertical pixels, respectively. The formula for calculating the *y*-coordinate of an aircraft within the apron sandbox’s scene coordinate system is given by Equation (1).

(1) $$\begin{array}{cc} y & =h_{c}\tan\left(\alpha +\beta \right)-d_{1} \end{array}$$

where:

(2) $$\begin{array}{cc} \beta & =\arctan\frac{d_{1}}{h_{c}} \end{array}$$

(3) $$\begin{array}{cc} \alpha & =\frac{1}{2}\tan^{-1}\frac{h_{c}d_{2}}{h_{c}^{2}+d_{1}^{2}+d_{1}d_{2}} \end{array}$$

The *x*-coordinate of an aircraft in the apron’s scene coordinate system is defined by Equation (4):

(4) $$x=\frac{\left(y\left(\frac{d_{1}}{2}-\frac{d_{3}}{2}\right)\frac{1}{d_{2}}+\frac{d_{1}}{2}\right)\left(x_{a}-p_{v}\right)}{p_{v}}$$

where: $p_{v}$ denotes the pixel width of the image.

### 2.3. Conflict Detection Model Based on Wingtip Keypoint Detection

Currently, regulations regarding wingtip clearance are based solely on standards set by the International Civil Aviation Organization (ICAO), which specify the minimum distance between an aircraft and another aircraft in a different parking position [18]. However, these regulations do not account for potential conflicts that may arise during aircraft operations, whether stationary or in motion. This paper proposes a novel delineation of wingtip alert zones, designed to address potential conflicts both when aircraft are parked and while they are in motion. By proactively identifying and mitigating risks associated with insufficient wingtip clearance, this approach aims to enhance safety protocols in dynamic airport environments.

**Figure 2:**
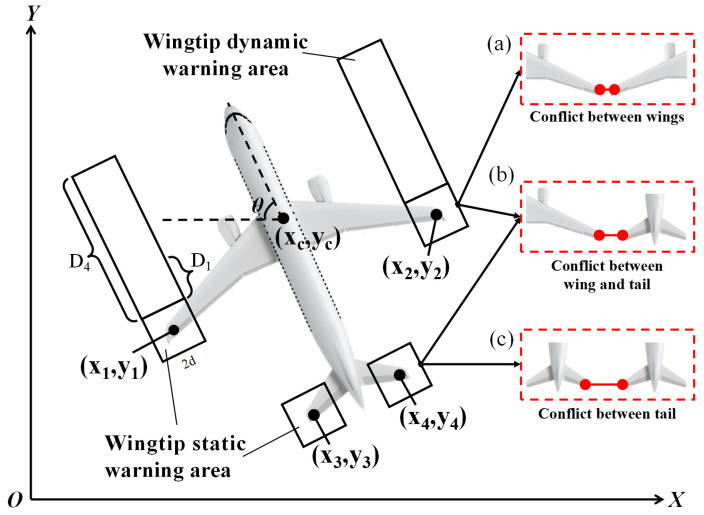
Wingtip dynamic warning area and possible conflict situations. (**a**) Conflict between wings, (**b**) conflict between wing and tail, (**c**) conflict between tails.

To achieve this, we divide wingtip alert zones into two categories: static and dynamic alert zones. As shown in Figure 2, the static alert zone is a square with a side length equal to twice the minimum clearance distance. The dynamic alert zone is designed based on the static alert zone and includes a rectangular area, the length of which is equivalent to the total braking distance. When delineating the dynamic alert zone, it is crucial to consider both the aircraft’s kinematic model and the impact of human factors on pilots. The total braking distance includes both the reaction distance and the braking distance. Previous studies have shown that pilots’ reaction times vary [19]. In this study, the maximum reaction time *t* for pilots is set to 1.5 s.

The reaction distance $D_{1}$, which represents the distance traveled during the reaction time, is calculated as follows:

(5) $$D_{1}=v_{0}t_{0}$$

where $v_{0}$ represents the aircraft’s taxiing speed during the reaction time, measured in meters per second (m/s), and $t_{0}$ denotes the reaction time, measured in seconds (s).

The braking process of an aircraft consists of two phases: the initial braking phase and the sustained braking phase. The initial braking distance is further divided into the brake system’s response process and the process of escalating braking to a certain level. During this phase, the aircraft sequentially undergoes uniform linear motion followed by decelerated motion.

Assuming that deceleration is linearly related to time during the experiment, the distance $D_{2}$ covered by the aircraft in the initial braking phase is calculated as follows:

(6) $$D_{2}=v_{0}t_{1}+v_{0}t_{2}-\frac{1}{2}a_{1}t_{2}^{2}$$

Here, $t_{1}$ denotes the brake system’s reaction time after initiating braking, measured in seconds (s). Meanwhile, $t_{2}$ is the time required for the brake system to reach a specific braking level, also measured in seconds (s). The parameter $a_{1}$ represents the aircraft’s deceleration once the brake pedal is engaged, expressed in meters per second squared (m/$s^{2}$).

During the sustained braking phase, a constant braking force is maintained until the aircraft comes to a complete stop, resulting in uniform deceleration. The distance covered in this phase is referred to as $D_{3}$.

(7) $$D_{3}=\frac{v_{0}^{2}}{2a_{1}}-\frac{1}{2}a_{1}t_{2}^{2}$$

According to the Federal Aviation Administration (FAA) guidelines on apron taxiing and braking tests [20], an aircraft’s deceleration efficiency primarily depends on the type of braking system and the runway surface conditions. In this work, we assume an anti-skid braking system operating on a dry runway, where the braking efficiency is characterized by two key factors: the baseline brake efficiency coefficient $C_{1}$ and the maximum friction coefficient $C_{2}$. Therefore:

(8) $$a_{2}=C_{1}\;g\;C_{2},$$

where *g* is the gravitational acceleration $\left(m/s^{2}\right)$.

The total braking distance is given by

$$D_{1}+D_{2}+D_{3}=v_{0}\;\left(t_{0}+t_{1}+t_{2}\right)+\frac{v_{0}^{2}}{2a_{1}}+\frac{v_{0}^{2}}{2a_{2}}-a_{1}\;t_{2}^{2}$$

Since $t_{2}$ is very small, the term $a_{1}\;t_{2}^{2}$ can be neglected, simplifying the formula for the total braking distance to:

(9) $$D_{4}=v_{0}\;\left(t_{0}+t_{1}+t_{2}\right)+\frac{v_{0}^{2}}{2}\;\left(\frac{a_{1}+a_{2}}{a_{1}\;a_{2}}\right).$$

Additionally, to determine the direction of the aircraft for delineating the dynamic warning zone, we calculate θ using Equation (10):

(10) $$\theta =\tan^{-1}\;\left[\frac{\frac{x_{1}+x_{2}}{2}\;-\;\frac{x_{3}+x_{4}}{2}}{\frac{y_{1}+y_{2}}{2}\;-\;\frac{y_{3}+y_{4}}{2}}\right].$$

To further enhance the functionality of the dynamic warning area, we divide it into two warning levels. The first level reminds airport controllers to closely monitor the target’s subsequent behavior to identify potential risks in a timely manner, while the second level requires additional measures, such as promptly notifying pilots to ensure safety. The level division is determined by:

(11) $$Level=\left\{\begin{array}{cc} 1, & \mathrm{if}\;\mathrm{Distance}<D_{1}+d,\\ 2, & \mathrm{if}\;\mathrm{Distance}\geq D_{1}+d. \end{array}\right.$$

As shown in Figure 2, the following describes potential conflict scenarios:

(1) **Two stationary aircraft (Figure 2a):** The detection system automatically identifies the target boxes and keypoints of each aircraft, displaying the static alert zones around their wingtips. It locates the pair of wingtips closest to each other. If these static alert zones overlap, the system immediately issues an alert signal; if they do not, no alert is triggered.
(2) **One or both aircraft in motion (Figure 2b,c):** The system computes aircraft speed based on each aircraft’s motion trajectory. If the gap between wingtips (or between a wingtip and a horizontal stabilizer tip) is shorter than the sum of the minimum safe distance, the distance covered during the reaction time at the current speed, and the full stopping distance, no alert is triggered. Conversely, when wingtip distances fall below the combined reaction and total braking distances, an alert is issued.

## 3. LSCD-Pose Algorithm

### 3.1. LSCD-Pose Algorithm

**Figure 3:**
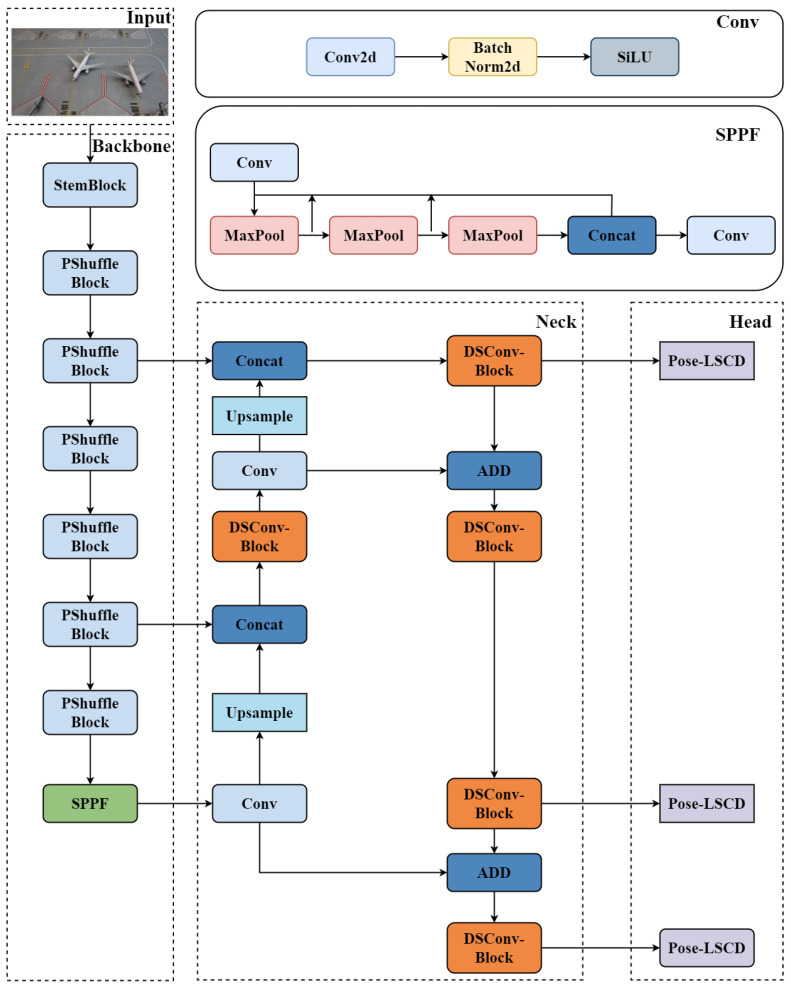
LSCD-Pose algorithm structure diagram.

In this paper, we introduce Lightweight Shared Convolutional Pose Detection (LSCD-Pose), an algorithm optimized through enhancements to the model’s backbone, neck, and head. The resulting LSCD-Pose architecture is illustrated in Figure 3.

### 3.2. Pose Estimation Algorithm Based on YOLO

The original You Only Look Once (YOLO) algorithm profoundly influenced the field of computer vision. Building on this foundation, researchers have successively developed a range of classic versions. Among these, YOLOv8—released by Ultralytics on 10 January 2023—offers higher detection accuracy and speed compared to previous iterations like YOLOv5 and YOLOv7. More recently, variants such as YOLOv9 [21], YOLOv11 [22], and YOLOv12 [23] have emerged, each aiming to further refine detection precision and efficiency. We include these versions in our experiments for a comprehensive performance comparison.

Pose estimation is a pivotal task in computer vision that involves precisely identifying specific points, or “keypoints”, in an image. These keypoints mark the critical details of an object, indicating essential segments such as joints, landmarks, or other defining features that characterize its shape and posture. Usually represented as 2D [x,y] or extended 3D [x,y,visible] coordinates, these points map the object’s spatial configuration within the image frame.

#### 3.2.1. StemBlock

**Figure 4:**
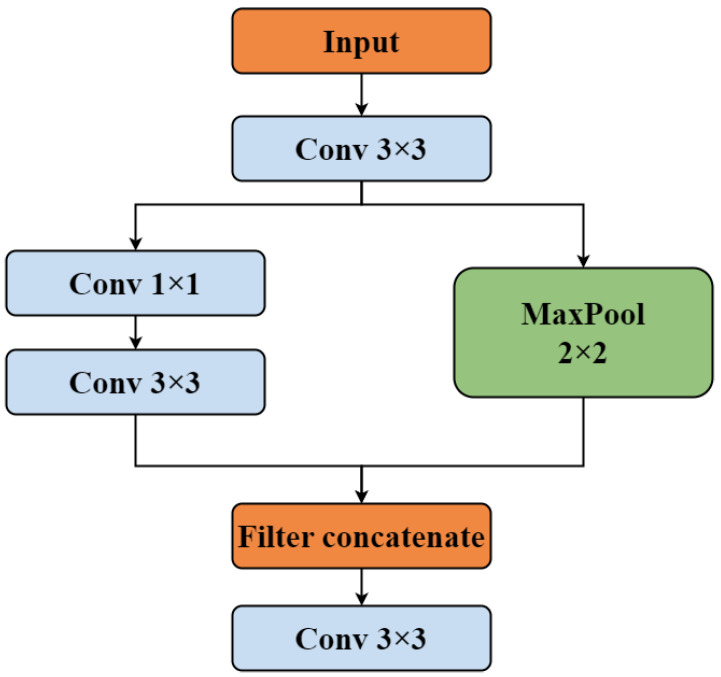
StemBlock structure diagram.

In the backbone, we first introduce the StemBlock structure [17]. StemBlock enhances feature representation capabilities without substantially increasing the number of parameters or computational complexity, thereby improving accuracy. The Filter concatenation technique mixes images of the same size by linking them in depth. The schematic diagram of the StemBlock structure is shown in Figure 4.

#### 3.2.2. PShuffle-Block

In our examination of the Shuffle-Block within ShuffleNetV2 [24], we observed that although the Rectified Linear Unit (ReLU) activation function works well during forward propagation, it can cause gradient vanishing when handling negative inputs during backpropagation. Furthermore, ReLU is not zero-centered, which leads to a bias shift in subsequent neural network layers and reduces the efficiency of gradient descent. Consequently, we adopt the non-linear Sigmoid Linear Unit (SiLU) as the activation function for ShuffleNet. The SiLU function is expressed as

(12) $$\mathrm{SiLU}\left(x\right)=x\times \mathrm{sigmoid}\left(x\right)=\frac{x}{1+e^{-x}},$$

where $\mathrm{sigmoid}\left(x\right)=\frac{1}{1+e^{-x}}$.

To further reduce parameters and enhance detection accuracy, we introduce Pinwheel Convolution (PSConv) [25]. Unlike conventional Convolution (Conv), PSConv employs asymmetric padding to generate horizontal and vertical convolution kernels for different regions of the image. Here, $h_{1}$, $w_{1}$, and $c_{1}$ refer to the height, width, and number of input channels, respectively. Batch Normalization (BN) and the SiLU activation function are applied after convolution to improve training stability and speed. The parallel convolution for the first layer of PSConv is defined as follows:

(13) $$\begin{array}{cc} X_{1}^{\left(h^{\prime },w^{\prime },c^{\prime }\right)} & =\mathrm{SiLU}\left(\mathrm{BN}(X_{P}^{\left(h_{1},w_{1},c_{1}\right)}⊗W_{1}^{\left(1,3,c^{\prime }\right)})\right),\\ X_{2}^{\left(h^{\prime },w^{\prime },c^{\prime }\right)} & =\mathrm{SiLU}\left(\mathrm{BN}(X_{P}^{\left(h_{1},w_{1},c_{1}\right)}⊗W_{2}^{\left(3,1,c^{\prime }\right)})\right),\\ X_{3}^{\left(h^{\prime },w^{\prime },c^{\prime }\right)} & =\mathrm{SiLU}\left(\mathrm{BN}(X_{P}^{\left(h_{1},w_{1},c_{1}\right)}⊗W_{3}^{\left(1,3,c^{\prime }\right)})\right),\\ X_{4}^{\left(h^{\prime },w^{\prime },c^{\prime }\right)} & =\mathrm{SiLU}\left(\mathrm{BN}(X_{P}^{\left(h_{1},w_{1},c_{1}\right)}⊗W_{4}^{\left(3,0,1,0\right)})\right). \end{array}$$

In this context, the symbol ⊗ denotes the convolution operation. We define $W_{1}^{\left(1,3,c^{\prime }\right)}$ as a 1×3 convolution kernel with an output channel dimension $c^{\prime }$. The padding parameter P(1,0,0,3) specifies left, right, top, and bottom padding. After the first interlaced convolution layer, the output feature map has a height $h^{\prime }$, width $w^{\prime }$, and channel count $c^{\prime }$, related to the input dimensions by

(14) $$h^{\prime }=\frac{h_{1}}{s}+1,\;w^{\prime }=\frac{w_{1}}{s}+1,\;c^{\prime }=\frac{c_{2}}{4},$$

where $c_{2}$ is the total number of output channels in the final PSConv module, and *s* is the stride.

After the first interlaced convolution, multiple feature maps are produced and concatenated using the Cat(·) operator:

(15) $$X^{\prime }\left(h^{\prime },w^{\prime },4c^{\prime }\right)=\mathrm{Cat}\left(X_{1}\left(h^{\prime },w^{\prime },c^{\prime }\right),\;\ldots ,\;X_{4}\left(h^{\prime },w^{\prime },c^{\prime }\right)\right).$$

Next, a convolution kernel $W^{\left(2,2,c_{2}\right)}$ is applied to the concatenated tensor without extra padding. To maintain compatibility with standard convolution layers and enable channel-attention mechanisms based on different convolution directions, we adjust the output feature map’s height and width to $h_{2}$ and $w_{2}$, respectively:

(16) $$h_{2}=h^{\prime }-1=\frac{h_{1}}{s},$$

(17) $$w_{2}=w^{\prime }-1=\frac{w_{1}}{s}.$$

Therefore, the final output is

(18) $$Y\left(h_{2},w_{2},c_{2}\right)=\mathrm{SiLU}\left(\mathrm{BN}\left(X^{\prime }\left(h^{\prime },w^{\prime },4c^{\prime }\right)⊗W^{\left(2,2,c_{2}\right)}\right)\right).$$

**Figure 5:**
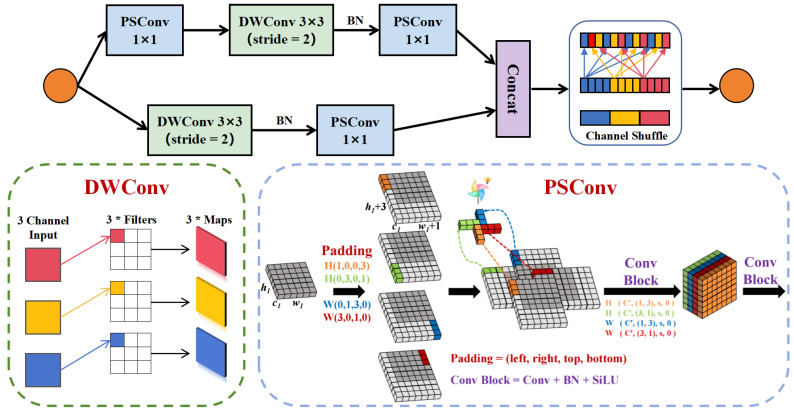
Schematic diagram of PShuffle-Block structure.

PShuffle-Block is illustrated in Figure 5.

#### 3.2.3. DSConv

**Figure 6:**
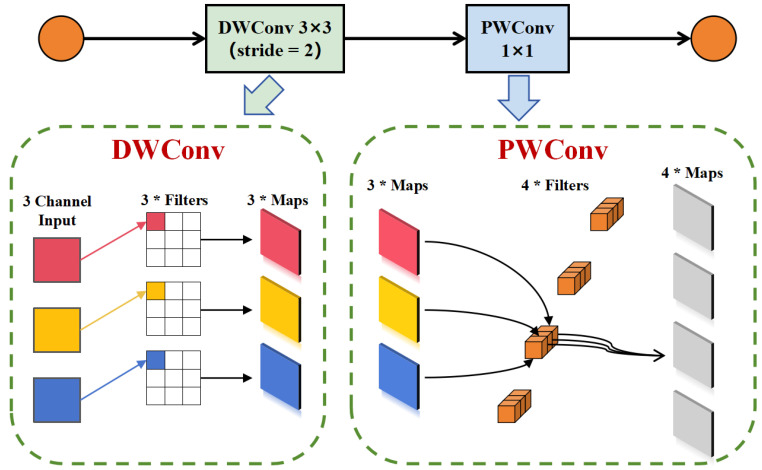
Schematic diagram of DSConv-Block structure

For the neck structure, we propose a novel neck configuration. Initially, we incorporated the Depthwise Separable Convolution (DSConv) block [26] structure and enhanced it by changing the activation function. The schematic diagram of this structure is shown in Figure 6.

The first convolution within the structure is the Depthwise Convolution (DWConv), which differs from standard convolutions in that it uses single-channel filters, requiring convolution operations on each input channel. This approach produces an output feature map with the same number of channels as the input feature map. In other words, the number of input feature map channels is equal to the number of convolution kernels, which is also equal to the number of output feature maps. This is followed by batch normalization and the SiLU activation function, then by Pointwise Convolution (PWConv), which operates like regular convolution but with a kernel size of 1×1×C. The 1×1 convolution does not alter the scale of the input tensor but can serve to increase or decrease its dimensionality.

The ratio of the number of parameters in depthwise separable convolution to that in standard convolution is:

(19) $$\frac{P_{D}}{P_{C}}=\frac{D_{k}\times D_{k}\times M+M\times N}{D_{k}\times D_{k}\times M\times N}=\frac{1}{N}+\frac{1}{D_{k}^{2}}$$

The size of the depthwise convolution kernel is $D_{k}\times D_{k}\times 1$, with *M* representing the number of kernels. The size of the pointwise convolution kernel is 1×1, with *N* representing the number of kernels. The computational ratio of depthwise separable convolution to standard convolution is as follows:

(20) $$\begin{array}{cc} \theta_{1} & =\frac{D_{k}\times D_{k}\times M\times D_{F}\times D_{F}}{D_{k}\times D_{k}\times M\times N\times D_{F}\times D_{F}} \end{array}$$

(21) $$\begin{array}{cc} \theta_{2} & =\frac{M\times N\times D_{F}\times D_{F}}{D_{k}\times D_{k}\times M\times N\times D_{F}\times D_{F}} \end{array}$$

(22) $$\begin{array}{cc} \frac{C_{p}}{C_{c}} & =\theta_{1}+\theta_{2}=\frac{1}{N}+\frac{1}{D_{k}^{2}} \end{array}$$

where $D_{F}$ is the spatial width or height of the feature map.

#### 3.2.4. LSCD-Pose

**Figure 7:**
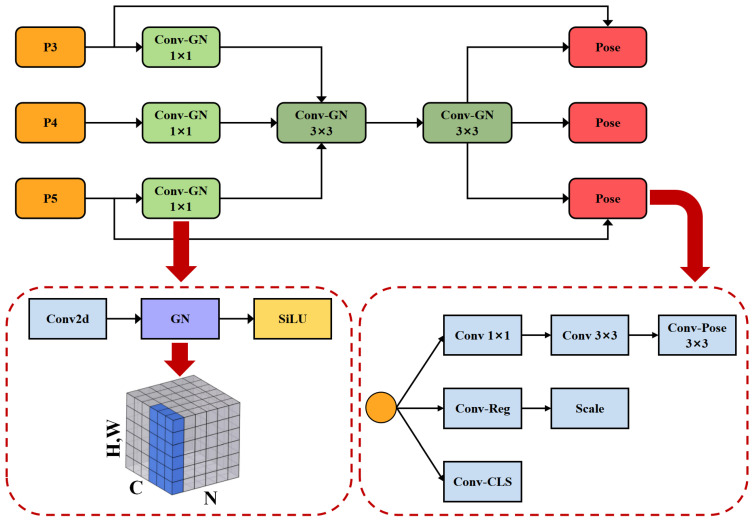
Schematic diagram of the LSCD-Pose structure.

As shown in Figure 7, we propose a novel Lightweight Shared Convolutional Detection Head, LCSD-Pose, which incorporates shared convolution layers to reduce parameter count and computational overhead while maintaining near-lossless detection accuracy. A key innovation of LSCD-Pose is the integration of Group Normalization (GN) [27] into the shared convolutional structure, leveraging GN’s robustness for both localization and classification [28]. Unlike Batch Normalization (BN), which relies on large mini-batches, GN normalizes features at the group level, thereby accelerating convergence and ensuring stable training across varying batch sizes. By grouping features with similar statistical distributions, GN facilitates effective gradient propagation through multiple convolutional kernels. Furthermore, the shared-convolution design significantly reduces redundant parameters, making LSCD-Pose particularly suitable for resource-constrained scenarios. Finally, a dedicated *scale* layer is introduced to adjust feature magnitudes, ensuring robust performance across a wide range of object scales. This head architecture achieves a favorable trade-off between efficiency and detection precision.

Overall, our LSCD-Pose design enables the detection head to maintain high accuracy while reducing the number of parameters and computational complexity.

## 4. Results and Analysis

### 4.1. Experimental Setup

To verify the effectiveness of the proposed method, we conducted our experiments on an Ubuntu 20.04 system using PyTorch 2.2.0 and Python 3.9. The hardware configuration included an Intel Xeon Platinum 8372C CPU (3.20 GHz) and an NVIDIA A800 Tensor Core GPU with 80 GB of PCIe memory. We set the learning rate to 0.01, used an image size of 1280×1280, and selected 0.937 for the momentum. Stochastic Gradient Descent (SGD) was employed as the optimizer over 210 epochs, with a batch size of 256. These parameters were chosen to balance computational demands and achieve optimal model performance.

### 4.2. Dataset and Evaluation Criteria

#### 4.2.1. Dataset

The experimental dataset in this study is derived from the surveillance video system of Ordos International Airport. A total of 2000 images were extracted, each containing annotations for aircraft, people, and vehicles. Initially, an image editing tool was used to crop and uniformly rename the images, ensuring their horizontal and vertical dimensions remained within 960 and 1080 pixels, respectively. Labelme was then employed for manual annotation, categorizing objects into three classes: aircraft, people, and vehicles. illustrates the annotation process. A top-down approach was used, first annotating detection boxes and then keypoints. After completing these annotations, the dataset was split into 1600 training samples, 200 validation samples, and 200 test samples, maintaining an 8:1:1 ratio.

#### 4.2.2. Evaluation Criteria

In evaluating the conflict detection algorithm and model based on wingtip keypoint detection, we employ multiple criteria to capture performance. Specifically, we employ the following:

- **Mean Average Precision (mAP)**: Assesses the accuracy of the algorithm. Its calculation is given in Equation (26). In the experiments, all models exhibited saturated performance under the mAP@50 metric. Therefore, we adopted mAP@50–95 as the primary accuracy evaluation metric to provide a more comprehensive and discriminative assessment of detection performance.
- **Frames Per Second (FPS)**: Reflects the speed of detection by counting the number of frames processed per second.
- **Parameters**: Indicates the total number of trainable parameters in the network.
- **Giga FLOPs (GFLOPS)**: Represents the computational complexity in billions of floating-point operations per second.
- **Detection Success Rate**: Probability that the algorithm correctly detects the aircraft whenever it appears.
- **Alert Success Rate**: Probability of issuing a correct warning whenever a conflict scenario occurs.
- **False Positive Rate (FP)**: Proportion of incorrect detections among all predicted positives.
- **False Negative Rate (FN)**: Proportion of missed detections among all actual positives.

(23) $$\begin{array}{cc} P & =\frac{TP}{TP+FP}, \end{array}$$

(24) $$\begin{array}{cc} R & =\frac{TP}{TP+FN}, \end{array}$$

(25) $$\begin{array}{cc} AP & =\int_{0}^{1}P\left(R\right)\;dR, \end{array}$$

(26) $$\begin{array}{cc} mAP & =\frac{1}{n}\sum_{i=1}^{n}AP. \end{array}$$

### 4.3. PShuffleNet vs. ShuffleNet: A Performance Comparison

To assess the effectiveness of our proposed PShuffleNet module, we compared it with the ShuffleNetv1-Block and ShuffleNetv2-Block baselines. As shown in Table 1, adopting the ShuffleNetv1-Block reduces the module size to 1.5 M and the parameters to 0.29 M, achieving a speed of 211.8 FPS—an improvement over the baseline’s 194.5 FPS. Similarly, using the ShuffleNetv2-Block decreases the computational load to 5.1 GFLOPs, though it yields a slightly lower FPS of 208.5. In contrast, our PShuffleNet module substantially increases the mAP@50–95 to 0.977 while maintaining a module size of 1.6 M and achieving the highest FPS of 217.1. These results demonstrate that PShuffleNet achieves an optimal balance between accuracy and inference speed, outperforming the other configurations.

### 4.4. Ablation Experiment

Table 2 summarizes the experimental results. When used independently, each module exhibits distinct trade-offs. StemBlock substantially reduces computational complexity (GFLOPs from 8.4 to 2.2) and doubles the FPS (194.5 to 388.8), but slightly lowers detection accuracy (mAP@50–95 drops from 0.975 to 0.933). PShuffle-Block notably decreases model size and parameters by 77.8% and 89.3%, respectively, moderately raises FPS by 11.6%, and slightly enhances accuracy (mAP@50–95 from 0.975 to 0.977). DSConv-Block significantly increases FPS by 49.5% and moderately reduces parameters and GFLOPs, with minimal impact on detection accuracy (mAP@50–95 remains at 0.974). LSCD-Pose alone provides balanced improvements by elevating FPS by 30.4%, cutting parameters and GFLOPs by about 23% and 20.2%, and marginally boosting accuracy to 0.976.

Combining two modules highlights additional efficiency-accuracy trade-offs. For instance, StemBlock and PShuffle-Block together greatly reduce parameters (90.3%) and GFLOPs (72.6%) while doubling FPS, but at the cost of accuracy (mAP@50–95 decreases to 0.936). However, pairing StemBlock with LSCD-Pose mitigates the accuracy loss (mAP@50–95 remains at 0.972) and still substantially improves FPS and lowers computational complexity.

Integrating three or more modules clarifies these trade-offs further. StemBlock, PShuffle-Block, and DSConv-Block combined yield the largest FPS increase (115.5%) and a notable drop in complexity but also a marked accuracy reduction (mAP@50–95 falls to 0.903). Adding LSCD-Pose to this mix recovers detection accuracy (mAP@50–95 improves to 0.956), delivers the highest FPS (461.7), and achieves the most balanced solution. Hence, the strategic integration of all modules significantly enhances efficiency and inference speed with minimal compromise in detection accuracy.

**Figure 8:**
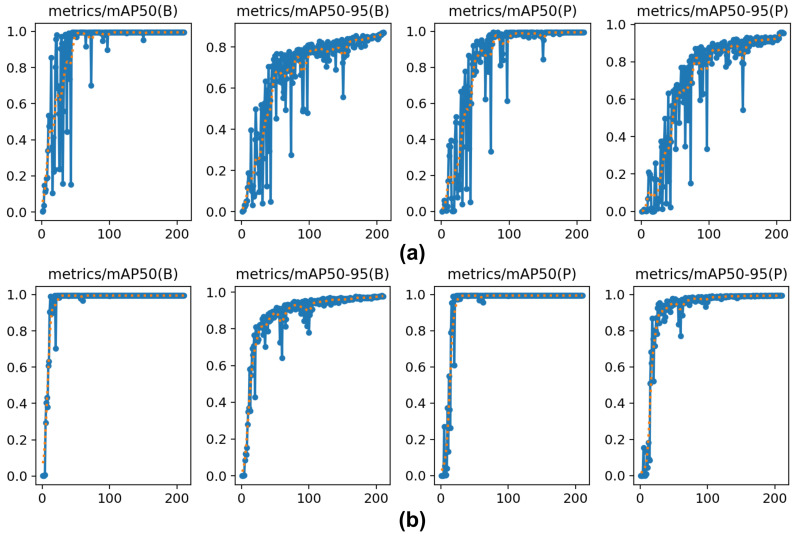
Comparison of the improved YOLOv8-Pose algorithm and the original algorithm. (**a**) YOLOv8-Pose, (**b**) Ours.

Analyzing the mAP of the improved algorithm relative to the original algorithm, as shown in Figure 8, reveals significant enhancements in multiple metrics. The improved algorithm converges more rapidly to optimal performance, suggesting a more efficient learning process—likely owing to a refined architecture or improved training methods. Such rapid convergence is particularly advantageous in scenarios requiring quick model deployment. Moreover, the improved algorithm demonstrates increased stability, with reduced accuracy fluctuation across iterations. This reliability is beneficial for handling diverse data variations. Most notably, the improved algorithm achieves higher accuracy for both object detection and keypoint identification, as reflected by its superior mAP@50 and mAP@50–95 scores. This precision is crucial for applications reliant on accurate detections and keypoint mappings, ensuring that subsequent analyses or actions are based on trustworthy data.

### 4.5. Performance Comparison of Different Keypoint Algorithms

In this work’s experimental section, we compare varying sizes of the YOLOv8-Pose and YOLOv9-Pose models. By examining mAP50–95, model size, parameter counts, GFLOPs, and FPS, we gauge each model’s performance across both accuracy and efficiency (Table 3).

Beyond these YOLO-based models, we evaluate multiple keypoint algorithms by focusing on accuracy, model size, parameter count, computational complexity, and FPS. As shown in Table 4, most algorithms, apart from Srcnet and YOLO-Pose, achieve accuracies exceeding 0.95. For model size and parameter counts, our improved algorithm yields the smallest values, making it highly suitable for embedded applications, while its computational complexity is marginally higher than that of ShuffleNetV1, ShuffleNetV2, or Lightweight High-Resolution Network (LiteHRNet), our algorithm delivers superior FPS performance.

This comparison underscores the efficiency of our improved algorithm, maintaining robust accuracy alongside operational efficacy—critical for real-time applications that demand both reliability and minimal resource use. Despite its slightly greater computational overhead, the high FPS indicates strong real-time inference, essential for scenarios such as autonomous systems, live surveillance, and interactive environments. Moreover, the reduced model size and parameter count further highlight its suitability for hardware-constrained deployments.

**Figure 9:**
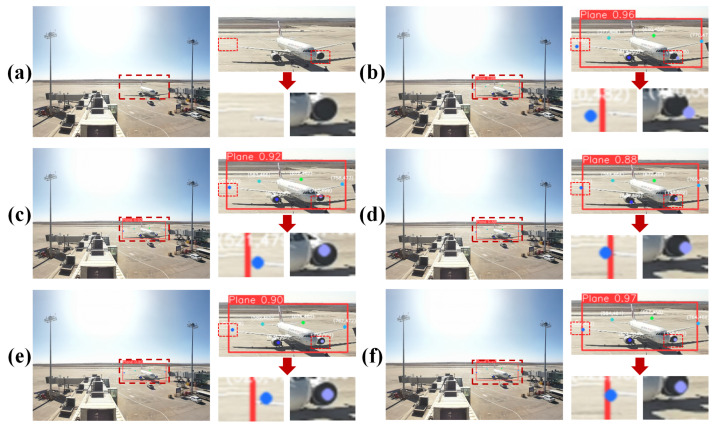
Inference results for different algorithms. (**a**) Original, (**b**) YOLOv8-Pose, (**c**) YOLOv9-Pose, (**d**) HRNet, (**e**) RTMPose, (**f**) Ours.

Under identical training parameters, we compared predictions from five different algorithms against the original annotated images (Figure 9). The results reveal varying levels of accuracy. In YOLOv8-Pose, the left wingtip and right engine show noticeable keypoint deviation from their respective bounding boxes. HRNet exhibits a similar issue, with the left wingtip keypoint extending beyond the target box, while YOLOv9-Pose and RTMPose do not demonstrate keypoint misalignment outside the bounding box, their bounding-box confidence scores fall below 0.95. By contrast, our algorithm locates wingtip keypoints accurately without deviation and consistently performs well for other components. This precision and reliability in detecting critical aircraft points considerably bolsters the overall effectiveness of the monitoring system.

### 4.6. The Impact of Different Road Surface Types and Braking Conditions on Performance

We also investigated the impact of different road conditions and braking types on system performance [39]. Changes in these factors alter the threshold of the dynamic warning area, consequently affecting detection and warning outcomes. Four scenarios were designed for this evaluation:

- **Scenario 1**: Wet road surface with non-anti-skid braking;
- **Scenario 2**: Dry road surface with non-anti-skid braking;
- **Scenario 3**: Wet road surface with anti-skid braking;
- **Scenario 4**: Dry road surface with anti-skid braking.

**Figure 10:**
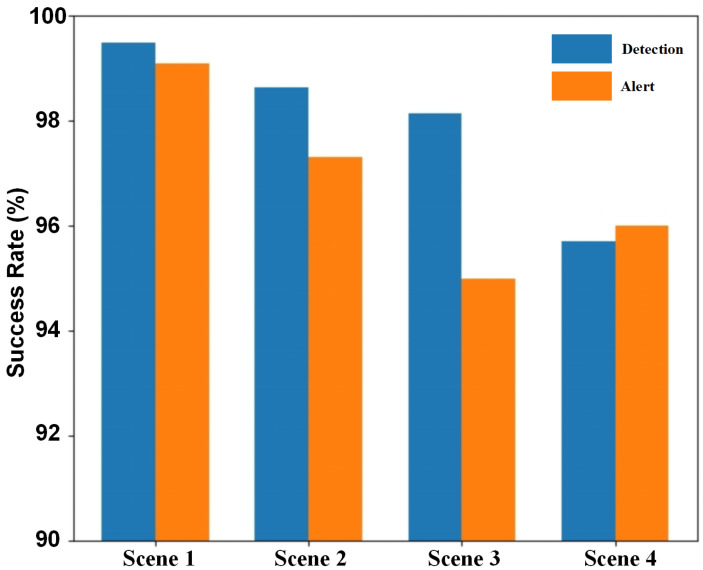
The impact of different road surface types and braking conditions on performance.

Table 5 presents the corresponding braking efficiency and adhesion coefficients for each condition. Experiments were conducted under all four scenarios, and the results are shown in Figure 10. It can be observed that as the parameter $a_{3}$ increases, both the detection and warning success rates exhibit a slight decline. This occurs because higher braking efficiency and adhesion coefficients reduce the size of the dynamic warning area when $a_{3}$ increases, thereby slightly lowering the warning success rate.

### 4.7. Visualization of Conflict Detection Model Results

**Figure 11:**
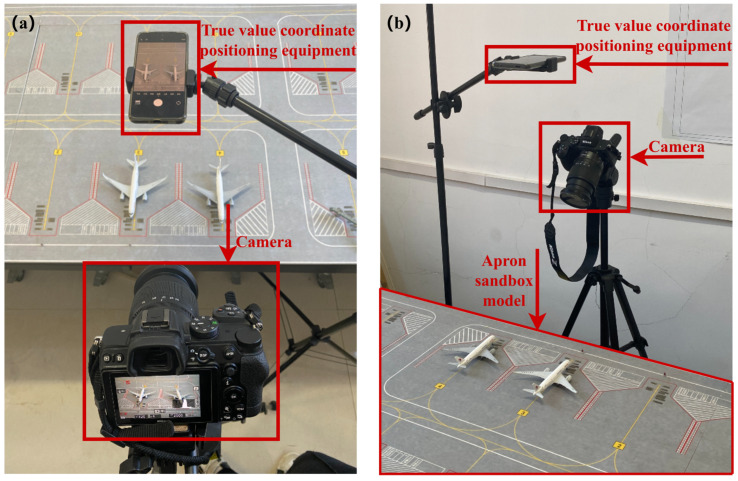
A diagram of camera calibration technology. (**a**), side view (**b**) top view.

Because collecting conflict image data in real-world scenarios is challenging, we employed an apron sandbox model to validate our model’s conflict detection effectiveness. A camera was placed in front of the sandbox to capture the images used for training and to record test videos simulating real airport operations. Additionally, a ground-truth coordinate device was installed above the sandbox to record the aircraft’s apron coordinates, which were compared against the coordinates converted by the model during experiments. This comparison is essential to confirm accuracy in real-world scenarios, as precise position tracking is critical for preventing conflicts and managing apron operations efficiently. By aligning the model’s output with the ground-truth data, we can identify and address any discrepancies, thereby enhancing the model’s reliability and overall effectiveness. Figure 11 illustrates the apron sandbox model and associated equipment.

In the first scenario, one aircraft is parked on the apron while another deviates from its intended taxi path as it approaches a nearby parking position. This deviation leads to a conflict between the taxiing aircraft’s wingtip and the stationary aircraft’s tail wingtip. In the second scenario, an aircraft is parked on the apron while another aircraft is taxiing along the centerline of its designated parking position. However, due to a deviation from the intended path, a wingtip-to-wingtip conflict occurs between the two aircraft.

**Figure 12:**
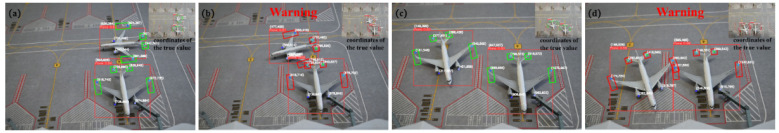
The experimental run diagram. (**a**) Scene 1, system does not warn, (**b**) Scene 1, system warns of wingtip clashes, (**c**) Scene 2, system does not warn, (**d**) Scene 2, system warns of wingtip clashes.

As shown in Figure 12, the detection outcome is displayed in green when no conflict is detected, and it turns red when a conflict occurs. These results demonstrate that our model can accurately identify both conflict and non-conflict scenarios.

To further evaluate our model, we recorded multiple video sequences in a simulated sandbox environment, towing the aircraft at various speeds. We then assessed the model using four metrics: Detection Success Rate, False Positives, False Negatives, and Alert Success Rate.

As shown in Table 6, the experimental results indicate that the model maintains high performance under different speed conditions. Nevertheless, as speed increases, each metric exhibits a slight decline, likely due to motion blur, which adversely affects detection accuracy.

## 5. Conclusions and Challenges

This paper investigates the impact of collaborative sensing on airport aircraft safety, underscoring its potential to enable real-time information sharing and effective decision-making. We present a novel coordinate conversion method that efficiently maps pixel coordinates to real-world coordinates, enhancing the accuracy of aircraft position and speed estimation. Additionally, we refine the YOLOv8-Pose algorithm, reducing model size and complexity while maintaining a high frame rate of 461.7 FPS. By tailoring both static and dynamic alert zones to aircraft motion states, our proposed model demonstrates robust real-time capabilities and accuracy in multiple experimental scenarios.

Despite these promising results, certain performance constraints should be noted, particularly under extreme weather conditions such as heavy rain, fog, or snow. These environmental factors can degrade sensor input quality, thereby affecting the precision of aircraft position estimation and real-time decision-making. Evaluating and ensuring the model’s reliability in these challenging conditions remain key topics for further research.

Furthermore, although collecting real conflict data is challenging due to the rarity of such events and the restrictions at operational airports, our training and validation relied on laboratory sandbox simulations. Future work will emphasize enhancing model robustness under diverse environmental contexts by simulating extreme weather scenarios and refining adaptation through advanced data augmentation. In addition, future improvements will focus on leveraging warning results under various weather conditions to optimize taxi routes, ensuring reliable and efficient support for air traffic controllers.

In summary, this research lays a foundation for optimizing taxi routes to mitigate collision risks and bolster airport safety margins. Our ongoing efforts aim to reinforce model robustness and environmental adaptability, thereby ensuring practical viability in real-world operations—even under adverse weather conditions.

## Figures and Tables

**Table 1:** Performance Comparison of Different Modules.

Module	mAP@50-95	Module Size (M)	Para (M)	GFLOPs	FPS
None	0.975	6.3	3.09	8.4	194.5
+ShuffleNetv1	0.963	1.5	0.29	6.7	211.8
+ShuffleNetv2	0.974	1.6	0.31	5.1	208.5
+PShuffleNet	**0.977**	1.6	0.33	4.7	**217.1**

**Table 2:** Ablation experiments with the modules.

StemBlock	PShuffle- Block	DSConv- Block	LSCD- Pose	mAP@50-95	Module Size	Params	GFLOPs	FPS
				0.975	6.3	3.09	8.4	194.5
√				0.933	6.2	3.09	2.2	388.8
	√			0.977	1.6	0.33	4.7	217.1
		√		0.974	3.9	1.84	6.3	290.7
			√	0.976	4.9	2.38	6.7	253.6
√	√			0.936	1.0	0.30	2.3	391.6
√			√	0.972	4.8	2.38	1.7	399.4
√	√	√		0.903	0.8	0.20	1.2	419.2
√	√		√	0.941	0.7	0.18	1.6	366.2
√	√	√	√	**0.956**	**0.6**	**0.11**	**1.5**	**461.7**

**Table 3:** Performance of different algorithms.

Module	mAP50-95	Module Size (M)	Para (M)	GFLOPs	FPS
YOLOv8n-Pose	0.975	6.3	3.09	8.4	194.5
YOLOv8s-Pose	**0.987**	23.3	11.41	29.4	101.1
YOLOv8m-Pose	0.981	53.4	26.40	80.8	39.5
YOLOv8l-Pose	0.983	89.5	44.59	168.5	23.7
YOLOv8x-Pose	0.986	139.6	69.45	263.2	13.1
YOLOv9u-Pose	0.986	65.4	3.23	122.4	46.9
YOLOv9c-Pose	0.986	53.5	26.30	107.4	21.9
YOLOv9e-Pose	0.981	119.1	59.00	196.4	17.5
Ours	0.956	**0.6**	**0.11**	**1.5**	**461.7**

**Table 4:** Performance of different keypoint detection algorithms.

Algorithms	mAP@50-95	Module Size (M)	Para (M)	GFLOPs	FPS
YOLO-Pose	0.947	3.9	14.35	20.40	69.52
YOLOv7-Pose	0.971	74.9	76.41	102.10	84.39
YOLOv8-Pose	0.975	6.3	3.09	8.40	194.5
YOLOv9-Pose	0.986	65.4	3.23	122.40	46.9
YOLOv11-Pose [22]	0.979	6.0	2.91	7.60	136.99
YOLOv12-Pose [23]	0.963	5.3	2.59	6.51	138.89
ShuffleNetV1 [29]	0.995	441.9	6.94	1.34	47.06
ShuffleNetV2 [24]	0.995	83.8	7.55	1.36	85.18
HRNet [30]	0.995	344.2	28.50	7.68	42.56
LiteHRNet [31]	0.997	23.6	1.76	0.43	50.86
ResNet50 [32]	0.989	408.9	34.00	5.44	5.64
ResNetv1d50 [33]	0.997	408.9	34.01	5.68	47.16
ResNext50 [34]	0.998	432.2	33.47	5.59	45.05
SeresNet [35]	0.995	441.9	36.53	5.45	44.31
HRformer [36]	0.997	522.9	43.21	14.10	11.5
Srcnet [37]	0.813	432.6	34.00	5.29	30.30
RTMPose [38]	0.995	212.4	13.17	1.90	275.85
Ours	0.956	**0.6**	**0.11**	**1.50**	**461.7**

**Table 5:** Braking Efficiency and Adhesion Coefficient under Different Conditions.

Pavement Surface/Brake Condition	Anti-Skid Brakes	Non-Anti-Skid Brakes
Dry pavement	$C_{1}$: 0.94/$C_{2}$: 0.8	$C_{1}$: 0.9/$C_{2}$: 0.3
Wet pavement	$C_{1}$: 0.81/$C_{2}$: 0.8	$C_{1}$: 0.81/$C_{2}$: 0.3

**Table 6:** Performance comparison under two scenarios.

Aircraft Speed (m/s)	Detection Success Rate (%)	False Positives (%)	False Negatives (%)	Alert Success Rate (%)
**Scene 1 (Single-Aircraft Motion)**				
0.5	99.13	1.02	0.51	98.51
1.0	98.77	1.39	0.80	98.21
1.5	98.02	1.51	1.03	97.80
**Scene 2 (Two-Aircraft Motion)**				
0.5	98.11	2.17	1.52	97.04
1.0	98.59	2.23	1.38	96.53
1.5	97.73	2.86	1.82	97.21

## Data Availability

The raw data supporting the conclusions of this article will be made available by the authors on request.
